# The Microbiota/Host Immune System Interaction in the Nose to Protect from COVID-19

**DOI:** 10.3390/life10120345

**Published:** 2020-12-11

**Authors:** Arianna Di Stadio, Claudio Costantini, Giorgia Renga, Marilena Pariano, Giampietro Ricci, Luigina Romani

**Affiliations:** 1 Department of Otolaryngology, University of Perugia, 06132 Perugia, Italy; giampietro.ricci@unipg.it; 2 Department of Medicine and Surgery, University of Perugia, 06132 Perugia, Italy; claudio.costantini@unipg.it (C.C.); giorgia.renga@unipg.it (G.R.); marilena.pariano@collaboratori.unipg.it (M.P.)

**Keywords:** COVID-19, microbiota, upper respiratory tract, tryptophan, aryl hydrocarbon receptor

## Abstract

Coronavirus disease 2019 (COVID-19) is caused by the severe acute respiratory syndrome coronavirus 2 (SARS-CoV-2) and is characterized by variable clinical presentation that ranges from asymptomatic to fatal multi-organ damage. The site of entry and the response of the host to the infection affect the outcomes. The role of the upper airways and the nasal barrier in the prevention of infection is increasingly being recognized. Besides the epithelial lining and the local immune system, the upper airways harbor a community of microorganisms, or microbiota, that takes an active part in mucosal homeostasis and in resistance to infection. However, the role of the upper airway microbiota in COVID-19 is not yet completely understood and likely goes beyond protection from viral entry to include the regulation of the immune response to the infection. Herein, we discuss the hypothesis that restoring endogenous barriers and anti-inflammatory pathways that are defective in COVID-19 patients might represent a valid strategy to reduce infectivity and ameliorate clinical symptomatology.

## 1. Introduction

Coronavirus disease 2019 (COVID-19), similarly to the flu virus, is transmitted through micro drops produced by sneezing, coughing, or simply speaking. The virus penetrates in the host through the upper airways, and the nasal barrier is the first defensive line to avoid infection [1]. Once the virus has entered target cells in the respiratory tract, the replication, maturation, and release of the virus occur while the host cell undergoes pyroptosis with the release of inflammatory molecules [2]. These initial events initiate an immune response that may lead to the resolution of the infection or, if dysfunctional, trigger a cytokine storm that exacerbates lung inflammation and increases the susceptibility to secondary bacterial or fungal infections [2]. In addition, the cytokine storm may cause potentially fatal multi-organ damage [2].

Host characteristics can influence the infectivity, severity, and outcomes of SARS-CoV-2 infection [3], and the local and systemic immune activities play a key role in the response to the virus aggression. Age ([4] and references therein) and gender [5,6] both impact on local and systemic immunity, but while the modulation of systemic immune responses can be quite easy and rapid to obtain, improving the local upper respiratory tract (URT) immune competence is slightly complex. In fact, age and gender determine substantial variation in the URT. Aging reduces the immune response capacity in the nasal mucosa and decreases muco-nasal clearance, a fundamental process to prevent virus infection. Furthermore, Di Stadio et al. [5] have shown that estrogen stimulation (hormone/gender effect) on the URT mucosa could reduce virus virulence thanks to the improvement of both nasal clearance and the local immune response. The authors speculated that this phenomenon could explain why women, despite affected as well as men, present less severe forms of infection with relative better outcomes (i.e., lower mortality).

Gender and age also affect the constituents of gut [7,8] and skin [9] microbiota, and we speculate that they can similarly affect the normal nasal flora [10]. Normal and healthy nasal flora is important to maintain a good balance in the upper airway tract [10,11] and can be helpful to contain and contrast viral infection [12]. Thus, therapies that affect the URT immune reactivity and microbiome composition and diversity may show promise in the treatment of a variety of human diseases, including respiratory infections. Up to date, despite the benefit shown on URT infection [13,14], the fine mechanisms through which local therapies with corticosteroids, antibiotics, and a variety of immunomodulators and nutraceuticals affect the local immune reactivity and microbial composition are largely unknown.

While alterations of the gut microbiota [15,16] and the lower respiratory tract [17] have been reported in COVID-19 patients, with potential diagnostic and therapeutic implications [18,19,20,21], the relationship between COVID-19 and the nasal microbiota has received little attention. Based on a previous hypothesis that emphasized the importance of the nasal barrier to fight COVID-19 [22], in this review we aim to discuss how immunomodulation could stimulate the local nasal immune response and empower the nasal microbiota to prevent SARS-CoV-2 penetration and virulence.

## 2. The Nose Ecosystem at Work: The Microbiota

The nose likely represents the major site of entry and infection by SARS-CoV-2. Indeed, most of inhaled air enters the body through the nose, and the nasal epithelium show the highest expression of the ACE2 coronavirus receptor [23,24]. The nose might also regulate the subsequent immune response and disease severity. For instance, stimulation of the nasal innate immune response by low doses of mouse hepatitis virus type 1 was able to prime lung immunity for protection against subsequent lethal SARS-CoV or influenza A virus infection [25]. The barrier function of the nasal mucosa as well as the regulation of the local and distal immune response are likely modulated by the microbiota, i.e., the communities of microorganisms that colonize all of the surfaces of the human body exposed to the external environment. The microbiota may act either locally or distally to modulate host physiological and pathological processes. For instance, in the so-called gut-lung axis, the microbiota of the gastrointestinal tract can cross-talk with the lung microbiota and both can influence the immune system to gauge the susceptibility of the host to respiratory infections [25]. Similar to other mucosal sites of the body, the nasal cavity also harbors a community of commensal microorganisms that likely represent an important player in mucosal homeostasis and protection against infection. 

Several studies have analyzed the composition of the nasal microbiome in healthy adult subjects. The major phyla belong to Actinobacteria and Firmicutes, followed by Proteobacteria and Bacteroidetes [11,26,27,28,29,30,31]. At the genus level, the nares are mainly colonized by lipophilic bacteria, such as Corynebacterium, Propionibacterium (Cutibacterium), and Staphylococcus, followed by other genera such as Moraxella, Streptococcus, and Dolosigranulum [28,29,30,31,32,33,34]. It is believed that commensal bacteria in the nasal cavity protect from opportunistic pathogens by competing for space and nutrients, and also by producing toxic molecules [35]. In addition, it has been recently shown that *Staphylococcus epidermidis*, which increases during nasal microbiome maturation in humans, stimulates the production of antimicrobial peptides by the nasal epithelium, which efficiently reduce pathogen colonization [36]. Moreover, *S. epidermidis* can also promote the production of interferon λ-dependent innate immunity by normal nasal epithelial cells to protect against the influenza virus [12], although interferon λ can in turn change the composition of the nasal microbiome and favor *Staphylococcus aureus* superinfection [37]. Although the interactions between the host, pathogens, and commensal bacteria are complex and still hard to decipher, it is becoming increasingly clear that dysbiosis in the nasal cavity, i.e., changes in the composition of the microbial communities, modulates the susceptibility of the host to pathological conditions, including, among others, acute respiratory tract infections [35]. 

Different microorganisms causing respiratory tract infections have been associated with changes in the nasal microbiome and include nonviral and viral pathogens. The former includes not only bacteria, such as *Streptococcus pneumoniae*, where it has been shown that IL-17 modulates the composition of the nasal microbiome to influence pneumococcal colonization [38], but also fungi. Indeed, we have recently characterized the microbiome of the URT of a large population of hematological patients and identified microbial signatures associated with invasive fungal infections (manuscript submitted), a major threat in this category of patients [39]. The associations between the nasal microbiome and viral infections have received more attention, although a causal relationship could not always be established. A recent study has evaluated the nasal and throat microbiomes in patients with influenza A and B infection and household contacts, and identified microbial signatures that could predict the risk of infection [40]. Similarly, it was shown that the nasal and throat microbiomes influenced the susceptibility to influenza infection [41] and were associated with influenza symptoms and duration of shedding in a household transmission study [42]. Importantly, the nasal microbiome cannot only influence the susceptibility to infection, but may also affect the efficacy of a live attenuated influenza vaccine [43], thus modulating its therapeutic efficacy. Similar associations with the nasal microbiome have been also demonstrated for respiratory syncytial virus bronchiolitis, with changes in the bacterial composition, alone [44] or in combination with host immune response [45], associated with the severity of symptoms, and the presence of specific taxa with the risk of recurrent wheezing after severe bronchiolitis [46]. Therefore, there may be shifts in the composition of the nasal microbiota that may result in pro- or anti-inflammatory patterns with effects on the susceptibility and course of infection. This paradigm has been elegantly demonstrated for the lung microbiome, in which the acellular bronchoalveolar lavage sample was distinguished in two patterns or pneumotypes, supraglottic predominant taxa (SPT) and background predominant taxa (BPT), based on the enrichment with taxa from the upper respiratory tract or the environment, respectively [47]. Notably, the pneumotype SPT was associated with increased lung inflammation and Th17 activation, and a blunted TLR4 response [47]. The presence of microbial patterns associated with distinct inflammatory responses has been also observed in the nasal microbiota. Healthy young adults were evaluated for their nasal microbiota before and at different times upon challenge with rhinovirus type 39 [48]. Upon grouping of the nasal microbiota into six clusters based on the predominant genera, it was demonstrated that the baseline nasal microbiota was associated with distinct nasal inflammatory responses, viral load, and symptom severity upon infection [48].

**Figure 1:**
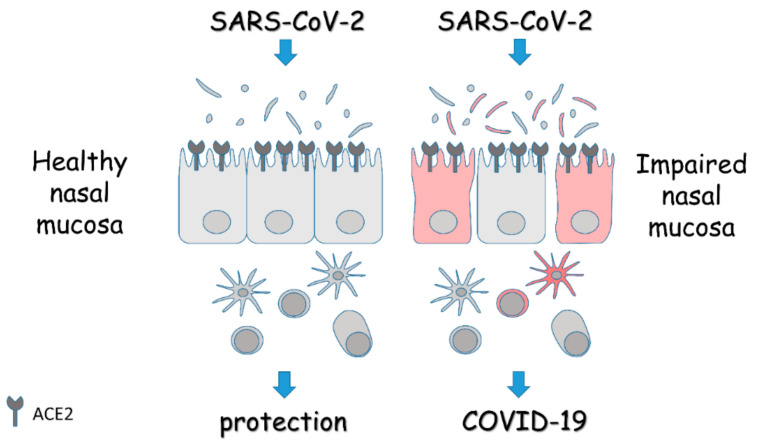
Schematic overview of the role of the nasal mucosa in COVID-19. The panel depicts the epithelial lining of the nasal mucosa expressing the entry receptors for SARS-CoV-2, and the associated microbiota and immune cells. In the presence of a healthy mucosa, the response to the virus is protective. On the contrary, in the presence of an impaired mucosa, the homeostasis is lost and alterations in microbiota, barrier integrity, and immune response turn the viral infection into overt disease. Details are described in the text.

While we are just beginning to understand the role of the nasal microbiota in bacterial, fungal, and viral infections, its involvement in SARS-CoV-2 infection has remained largely unexplored and may reveal peculiar properties that reflect the specific etiopathogenesis of COVID-19. A study comparing the nasopharyngeal microbiota of patients positive or negative for COVID-19 was recently published [49]. No differences were revealed in diversity or composition [49], although the small number of samples and the presence of potential confounders may have hindered the identification of distinct signatures. Nevertheless, accumulating evidence points to a role of dysbiosis in barrier impairment with increased susceptibility to infection and dysregulated immune response. In this regard, potential approaches aimed at restoring the mucosal homeostasis with direct activity on microbiome composition and/or modulation of the immune response may represent valuable options for the prevention of COVID-19, and the use of treatments not associated with side effects would be desirable to increase the efficacy/safety profile (Figure 1).

## 3. The Nose Ecosystem at Work: The Nasal Barrier

The nose serves important physiologic functions; it filtrates, warms, and humidifies the air so that pollution, viruses, and bacteria remain confined in the upper expiratory tract (URT), reducing the risk of inflammation and infection of the lungs [5,50]. The particular anatomy of the nose is specifically designed to promote nasal clearance by correctly driving the airflow from the external nostrils through the choana to the nasal posterior airways space (NPAS) [51,52]. There are three structures in each chona, called turbinates, that support the air passage and, thanks to the mucosa on their surface, further ameliorate nasal clearance [52]. The nasal choana is entirely coated with respiratory mucosa, consisting of a ciliated, highly vascularized, pseudostratified epithelium containing a sizeable number of mucus-producing goblet cells [51]. All these cells contribute to the fight against particles, viruses, and bacteria by working in synergy. In particular, the epithelial cells (ECs) provide a physical barrier and, working in conjunction with mucus glands and cilia, filter off the particles that enter the nose. Furthermore, ECs activate the local immune response, a fundamental step to block virus infiltration into the URT [5] and avoid the spread of infection in the pulmonary tract.

**Figure 2:**
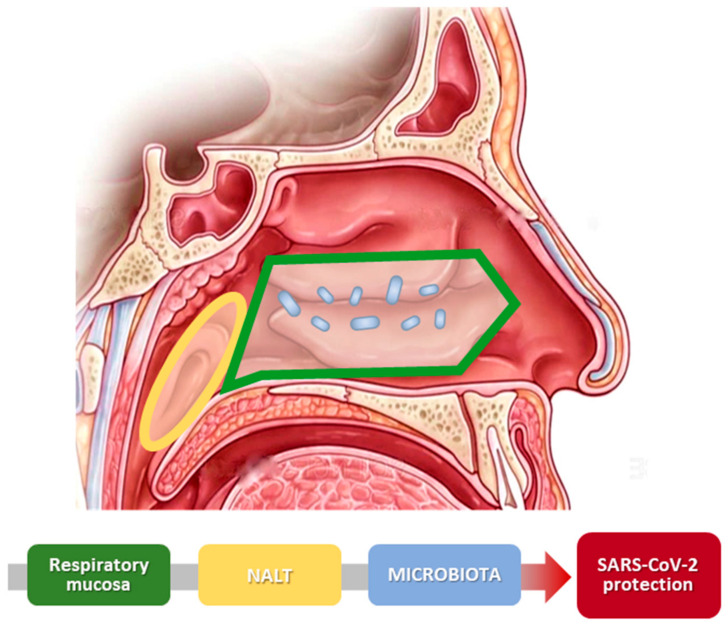
Scheme of nasal physiology and SARS-CoV-2. The image illustrates the different parts of the nose that create the nasal barrier. Nasal mucosa and nasal microbiota cooperate and improve the protection against virus infection.

Through the antigen-binding protein, ECs introduce and present the antigen to T-cells facilitating the immune response; this mechanism is also supported by ECs pro-inflammatory cytokines production, which improves the efficacy of the nasal local immunity [50]. As support of these cells, there is the action of the endothelial cells that attracts leukocytes to the site of inflammation [51] (Figure 2).

The final support to the nasal barrier is provided by the nasal-associated lymphoid tissue (NALT), which is widely diffused in children’s nose, but tends to disappear and be present only in the NPAS in the adult population [53]. 

Nasal microbiota can be a valid ally of the nasal immune response and helpful to fight viral and bacterial infection, as showed by Salzano et al. [54]. The authors showed that the nasal microbiota acts on the nasal immune response being responsible for the different responses that are observable in individuals suffering from nasal inflammation [54]. Specifically, the nasal microbiota is critical for the development of the mucosa-associated lymphoid tissue and the modulation of adaptive responses such as the production of IgA and the activity of T cells. Furthermore, nasal microbiota regulates the nasal epithelial barrier functions through the increased secretion of mucus and the control of paracellular transport across tight junctions [54].

## 4. Targeting the Nasal Microbiota-Immune System Cross-Talk

**Figure 3:**
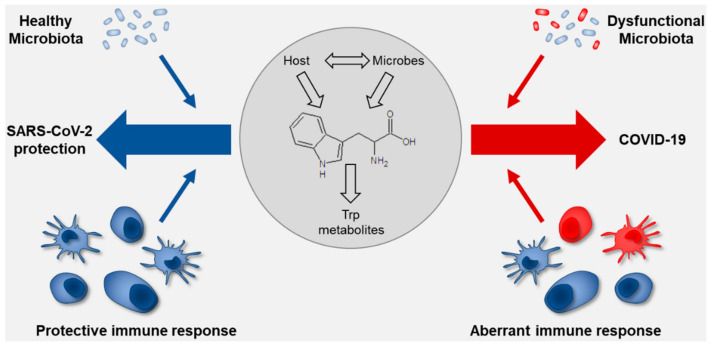
Schematic overview on the role of tryptophan metabolism at the interface of the microbiota and the immune system in SARS-CoV-2 infection. The drawing shows how the nasal mucosa and microbiota (healthy or dysfunctional) determine the outcome of infection. The healthy nasal microbiota positively interacts with the nasal mucosa improving the local immune response and allows the control of virus penetration with limited systemic effect. Conversely, a dysfunctional microbiota has a negative impact on the nasal mucosa, reducing the capacity of the nose to prevent the virus from causing overt disease. The tryptophan co-metabolism between the host and the microbiota may condition the response to viral infection and shift the balance between the different scenarios. Details are described in the text.

The cross-talk between the commensal microorganisms and the immune system plays a crucial role in mucosal homeostasis, including the interaction with pathogens and the outcome of infection (Figure 3).

The microbiota may protect from pathogens through several mechanisms, which include competition for nutrients, space, and production of anti-microbial peptides. At the same time, the microbiota instructs the immune system to be tolerant towards commensal microorganisms while being prepared to vigorously respond to pathogen colonization. This form of mucosal tolerance is crucial to maintain the balance between the myriad of microbial stimuli to which the immune system is exposed and to fine-tune the immune response based on the effective risk of infection. The delicate regulatory mechanisms that take place in the mucous membranes are susceptible to alterations that may result in pathological conditions, for instance, following microbial dysbiosis, disruption of the epithelial barrier integrity, or loss of the discriminatory function by the immune system. These general mechanisms described in mucous membranes, such as the intestine and the lung, most likely apply to the nasal mucosa [55], which is exposed to microbial or non-microbial environmental antigens constantly inhaled from the outside and interacting with the stable communities of microorganisms that populate the nasal cavities and the immune cells residing within the nasal-associated lymphoid tissue. Therefore, restoring mucosal homeostasis might represent a valuable strategy to protect from a variety of insults, including pathogen invasion. One such strategy would be represented by the intranasal instillation of bacterial species. For instance, intranasal *Staphylococcus epidermidis* administration protected from *Staphylococcus aureus* in a mouse model of sinusitis [56]. Similarly, intranasal inoculation of *Lactobacillus sakei* protected against *Corynebacterium tuberculostearicum* sinus infection in a murine model [57]. De Boeck et al. [34] demonstrated that lactobacilli were reduced in the upper respiratory tract of chronic rhinosinusitis patients and tested a formulation of *L. casei* AMBR2 for nasal administration in healthy volunteers for future therapeutic application. Besides probiotic administration, an alternative strategy would be represented by the modulation of nutrients upon which both the host and the microbiota converge for functional cross-regulation. One such nutrient is represented by tryptophan (Trp), an essential amino acid in mammals, which is the substrate of a multitude of host and microbial metabolic pathways in the generation of bioactive molecules [58,59,60]. The Trp metabolic pathways play a critical role in immune regulation and immune tolerance. In the host, Trp is catabolized in two major metabolic pathways for the production of kynurenines, via the rate-limiting enzymes indoleamine 2, 3-dioxygenase 1 (IDO1), IDO2, tryptophan 2, 3-dioxygenase, or serotonin [61]. IDO1 is a crucial regulator of the innate and adaptive immune system that acts by depleting Trp at the host/tumor/microbe interface and polarizing the immune response via the activation of regulatory T cells and myeloid-derived suppressor cells [59,62,63]. The potential role of the IDO1 pathway in COVID-19 has been recently described. A metabolomic study revealed that the kynurenine pathway was activated in COVID-19 patients [64]. In agreement with these findings, another study found that Trp metabolism was among the top pathways affected by COVID-19, with reduced levels of Trp, serotonin, and indole-pyruvate, and increased levels of kynurenine, kynurenic acid, picolinic acid, and nicotinic acid, but not anthranilate, suggesting hyperactivation of the kynurenine pathway [65]. 

The above results would suggest that restoring homeostatic Trp catabolism to re-equilibrate the generation of the different Trp metabolites may represent a therapeutic strategy in COVID-19. Indeed, the IDO1-dependent kynurenine can work as an agonist of the aryl hydrocarbon receptor (AhR), a ligand-activated transcription factor involved in a wide range of physiological processes, including regulation of the immune response [66]. AhR, in turn, may up-regulate the expression and/or activity of IDO1, thus generating a feed-forward loop with consequences on the immune response [59]. Based on the evidence that IL-6 down-regulates IDO1 activity, it has been hypothesized that the beneficial effects of tocilizumab, the blocking antibody against the IL-6 receptor, may also depend on the restoration of the IDO1-AhR pathway [67].

The potential role of the IDO1-AhR pathway is however disputed. A possible aberrant activation of AhR and IDO1 has been put forward to explain the symptomatology of COVID-19 patients [68], and a recent study identified AhR signaling as a common host response to infection by coronaviruses responsible for lung pathogenesis [69]. These results would suggest that inhibiting AhR activity could be therapeutically exploited in COVID-19. However, AhR is a promiscuous receptor that can bind multiple ligands with both positive and negative effects on inflammation, immunity, and infections [66]. In particular, microbial ligands working as AhR agonists may play a beneficial activity in the regulation of mucosal homeostasis, for instance by promoting epithelial barrier integrity [70,71,72], thus increasing the resistance to infection, and, in turn, AhR may regulate the composition of the microbiome [73]. Therefore, it remains to be seen whether AhR antagonism or agonism would be the most promising therapeutic strategy in COVID-19. Whatever the case, it is important to consider the Trp metabolism as a whole, thus including the host and microbial metabolic pathways, as alterations of the Trp flux may result in pathological conditions [60,74]. The balance between the IDO1 and AhR pathways represents a surrogate for the status of the microbiota and the local immune system and a predictive factor for the susceptibility to infection and the disease course. Therefore, the characterization of Trp metabolism in the nasal mucosa would provide information on the host–microbiota cross-talk and open a new avenue for intervention to maintain a balance between immune tolerance and colonization resistance.

## 5. Current Clinical Trials Targeting the Nasal Microbiota/Immune System in COVID-19 Patients

The concept of targeting the nasal microbiota/immune system in SARS-CoV-2 infection is currently being exploited in clinical trials. In trial NCT04347538, patients testing positive for COVID-19 are treated with saline nasal irrigation alone or with baby shampoo to reduce viral shedding and load. Changes in the mucosal immune response and microbial load in the nasopharinx as well as viral load will be measured as primary outcomes. The trial NCT04410263 enrolls COVID-19-positive patients admitted to ICU, and the microbiota will be evaluated, among other parameters, to identify risk factors for the development of acute respiratory distress syndrome, as a prerequisite for future therapeutic strategies. Finally, the clinical trial NCT04458519 is testing the use of intranasal probiotics in COVID-19-positive patients with mild-to-moderate symptoms, and the changes in the severity of infection will be evaluated as primary outcomes.

In conclusion, these ongoing clinical trials emphasize the rationale for targeting the nasal microbiota and immune system to prevent and/or treat SARS-CoV-2 infections, as discussed in the present review.

## 6. Conclusions

COVID-19 is still a major threat and, at the time of writing, more than 36 million people have been infected worldwide, with more than 1 million deaths. It is imperative to adopt strategies to control infectious rates and reduce the severity of the disease. Since the virus mainly enters the body through the upper airways, one such strategy would be to reinforce the mucosal barrier, which includes the epithelial lining, the local immune system, and the microbiota. The latter component of the triad can be considered a bona fide natural immune barrier and a target for intervention, not only to improve its intrinsic resistance to infection, but also to homeostatically regulate the local immune system.

## References

[1] Baron S., Fons M., Albrecht T. (1996). Viral Pathogenesis. Medical Microbiology.

[2] Tay M.Z., Poh C.M., Rénia L., MacAry P.A., Ng L.F.P. (2020). The trinity of COVID-19: Immunity, inflammation and intervention. Nat. Rev. Immunol..

[3] Di Stadio A., Ricci G., Greco A., de Vincentiis M., Ralli M. (2020). Mortality rate and gender differences in COVID-19 patients dying in Italy: A comparison with other countries. Eur. Rev. Med. Pharmacol. Sci..

[4] Mallapaty S. (2020). The coronavirus is most deadly if you are older and male-new data reveal the risks. Nature.

[5] Di Stadio A., Della Volpe A., Ralli M., Ricci G. (2020). Gender differences in COVID-19 infection. The estrogen effect on upper and lower airways. Can it help to figure out a treatment?. Eur. Rev. Med. Pharmacol. Sci..

[6] Scully E.P., Haverfield J., Ursin R.L., Tannenbaum C., Klein S.L. (2020). Considering how biological sex impacts immune responses and COVID-19 outcomes. Nat. Rev. Immunol..

[7] Yurkovetskiy L., Burrows M., Khan A.A., Graham L., Volchkov P., Becker L., Antonopoulos D., Umesaki Y., Chervonsky A.V. (2013). Gender bias in autoimmunity is influenced by microbiota. Immunity.

[8] Markle J.G., Frank D.N., Mortin-Toth S., Robertson C.E., Feazel L.M., Rolle-Kampczyk U., von Bergen M., McCoy K.D., Macpherson A.J., Danska J.S. (2013). Sex differences in the gut microbiome drive hormone-dependent regulation of autoimmunity. Science.

[9] Ying S., Zeng D.N., Chi L., Tan Y., Galzote C., Cardona C., Lax S., Gilbert J., Quan Z.X. (2015). The Influence of Age and Gender on Skin-Associated Microbial Communities in Urban and Rural Human Populations. PLoS ONE.

[10] Proctor D.M., Relman D.A. (2017). The Landscape Ecology and Microbiota of the Human Nose, Mouth, and Throat. Cell Host Microbe.

[11] Kumpitsch C., Koskinen K., Schopf V., Moissl-Eichinger C. (2019). The microbiome of the upper respiratory tract in health and disease. BMC Biol..

[12] Kim H.J., Jo A., Jeon Y.J., An S., Lee K.M., Yoon S.S., Choi J.Y. (2019). Nasal commensal Staphylococcus epidermidis enhances interferon-lambda-dependent immunity against influenza virus. Microbiome.

[13] Della Volpe A., Ricci G., Ralli M., Gambacorta V., De Lucia A., Minni A., Pirozzi C., Paccone M., Pastore V., Di Stadio A. (2019). The effects of oral supplements with Sambucus nigra, Zinc, Tyndallized Lactobacillus acidophilus (HA122), Arabinogalactans, vitamin D, vitamin E and vitamin C in otitis media with effusion in children: A randomized controlled trial. Eur. Rev. Med. Pharmacol. Sci..

[14] Di Stadio A., Della Volpe A., Korsch F.M., De Lucia A., Ralli M., Martines F., Ricci G. (2020). Difensil Immuno Reduces Recurrence and Severity of Tonsillitis in Children: A Randomized Controlled Trial. Nutrients.

[15] Gu S., Chen Y., Wu Z., Chen Y., Gao H., Lv L., Guo F., Zhang X., Luo R., Huang C. (2020). Alterations of the Gut Microbiota in Patients with COVID-19 or H1N1 Influenza. Clin. Infect. Dis..

[16] Zuo T., Zhang F., Lui G.C.Y., Yeoh Y.K., Li A.Y.L., Zhan H., Wan Y., Chung A.C.K., Cheung C.P., Chen N. (2020). Alterations in Gut Microbiota of Patients With COVID-19 During Time of Hospitalization. Gastroenterology.

[17] Shen Z., Xiao Y., Kang L., Ma W., Shi L., Zhang L., Zhou Z., Yang J., Zhong J., Yang D. (2020). Genomic Diversity of Severe Acute Respiratory Syndrome-Coronavirus 2 in Patients with Coronavirus Disease 2019. Clin. Infect. Dis..

[18] Akour A. (2020). Probiotics and COVID-19: Is there any link?. Lett. Appl. Microbiol..

[19] Iddir M., Brito A., Dingeo G., Fernandez Del Campo S.S., Samouda H., La Frano M.R., Bohn T. (2020). Strengthening the Immune System and Reducing Inflammation and Oxidative Stress through Diet and Nutrition: Considerations during the COVID-19 Crisis. Nutrients.

[20] Kalantar-Zadeh K., Ward S.A., Kalantar-Zadeh K., El-Omar E.M. (2020). Considering the Effects of Microbiome and Diet on SARS-CoV-2 Infection: Nanotechnology Roles. ACS Nano.

[21] Khatiwada S., Subedi A. (2020). Lung microbiome and coronavirus disease 2019 (COVID-19): Possible link and implications. Hum. Microbiome J..

[22] Ruggieri A., Anticoli S., D’Ambrosio A., Giordani L., Viora M. (2016). The influence of sex and gender on immunity, infection and vaccination. Annali Ist. Super. Sanita.

[23] Hou Y.J., Okuda K., Edwards C.E., Martinez D.R., Asakura T., Dinnon K.H., Kato T., Lee R.E., Yount B.L., Mascenik T.M. (2020). SARS-CoV-2 Reverse Genetics Reveals a Variable Infection Gradient in the Respiratory Tract. Cell.

[24] Sungnak W., Huang N., Becavin C., Berg M., Queen R., Litvinukova M., Talavera-López C., Maatz H., Reichart D., Sampaziotis F. (2020). SARS-CoV-2 entry factors are highly expressed in nasal epithelial cells together with innate immune genes. Nat. Med..

[25] Hua X., Vijay R., Channappanavar R., Athmer J., Meyerholz D.K., Pagedar N., Tilley S., Perlman S. (2018). Nasal priming by a murine coronavirus provides protective immunity against lethal heterologous virus pneumonia. JCI Insight.

[26] Bassis C.M., Tang A.L., Young V.B., Pynnonen M.A. (2014). The nasal cavity microbiota of healthy adults. Microbiome.

[27] Brugger S.D., Bomar L., Lemon K.P. (2016). Commensal-Pathogen Interactions along the Human Nasal Passages. PLoS Pathog..

[28] Bomar L., Brugger S.D., Lemon K.P. (2018). Bacterial microbiota of the nasal passages across the span of human life. Curr. Opin. Microbiol..

[29] Koskinen K., Reichert J.L., Hoier S., Schachenreiter J., Duller S., Moissl-Eichinger C., Schöpf V. (2018). The nasal microbiome mirrors and potentially shapes olfactory function. Sci. Rep..

[30] Rawls M., Ellis A.K. (2019). The microbiome of the nose. Ann. Allergy Asthma Immunol..

[31] Hardy B.L., Merrell D.S. (2020). Friend or Foe: Interbacterial Competition in the Nasal Cavity. J. Bacteriol..

[32] Man W.H., de Steenhuijsen Piters W.A., Bogaert D. (2017). The microbiota of the respiratory tract: Gatekeeper to respiratory health. Nat. Rev. Microbiol..

[33] Escapa I.F., Chen T., Huang Y., Gajare P., Dewhirst F.E., Lemon K.P. (2018). New Insights into Human Nostril Microbiome from the Expanded Human Oral Microbiome Database (eHOMD): A Resource for the Microbiome of the Human Aerodigestive Tract. mSystems.

[34] De Boeck I., van den Broek M.F.L., Allonsius C.N., Spacova I., Wittouck S., Martens K., Wuyts S., Cauwenberghs E., Jokicevic K., Vandenheuvel D. (2020). Lactobacilli Have a Niche in the Human Nose. Cell Rep..

[35] Dimitri-Pinheiro S., Soares R., Barata P. (2020). The Microbiome of the Nose-Friend or Foe?. Allergy Rhinol..

[36] Liu Q., Liu Q., Meng H., Lv H., Liu Y., Liu J., Wang H., He L., Qin J., Wang Y. (2020). Staphylococcus epidermidis Contributes to Healthy Maturation of the Nasal Microbiome by Stimulating Antimicrobial Peptide Production. Cell Host Microbe.

[37] Planet P.J., Parker D., Cohen T.S., Smith H., Leon J.D., Ryan C., Hammer T.J., Fierer N., Chen E.I., Prince A.S. (2016). Lambda Interferon Restructures the Nasal Microbiome and Increases Susceptibility to Staphylococcus aureus Superinfection. mBio.

[38] Ritchie N.D., Ijaz U.Z., Evans T.J. (2017). IL-17 signalling restructures the nasal microbiome and drives dynamic changes following Streptococcus pneumoniae colonization. BMC Genom..

[39] Pagano L., Busca A., Candoni A., Cattaneo C., Cesaro S., Fanci R., Nadali G., Potenza L., Russo D., Tumbarello M. (2017). Risk stratification for invasive fungal infections in patients with hematological malignancies: SEIFEM recommendations. Blood Rev..

[40] Tsang T.K., Lee K.H., Foxman B., Balmaseda A., Gresh L., Sanchez N., Ojeda S., Lopez R., Yang Y., Kuan G. (2019). Association between the respiratory microbiome and susceptibility to influenza virus infection. Clin. Infect. Dis..

[41] Lee K.H., Gordon A., Shedden K., Kuan G., Ng S., Balmaseda A., Foxman B. (2019). The respiratory microbiome and susceptibility to influenza virus infection. PLoS ONE.

[42] Lee K.H., Foxman B., Kuan G., Lopez R., Shedden K., Ng S., Balmaseda A., Gordon A. (2019). The respiratory microbiota: Associations with influenza symptomatology and viral shedding. Ann. Epidemiol..

[43] Salk H.M., Simon W.L., Lambert N.D., Kennedy R.B., Grill D.E., Kabat B.F., Poland G.A. (2016). Taxa of the Nasal Microbiome Are Associated with Influenza-Specific IgA Response to Live Attenuated Influenza Vaccine. PLoS ONE.

[44] Schippa S., Frassanito A., Marazzato M., Nenna R., Petrarca L., Neroni B., Bonfiglio G., Guerrieri F., Frasca F., Oliveto G. (2020). Nasal Microbiota in RSV Bronchiolitis. Microorganisms.

[45] Sonawane A.R., Tian L., Chu C.Y., Qiu X., Wang L., Holden-Wiltse J., Grier A., Gill S.R., Caserta M.T., Falsey A.R. (2019). Microbiome-Transcriptome Interactions Related to Severity of Respiratory Syncytial Virus Infection. Sci. Rep..

[46] Mansbach J.M., Luna P.N., Shaw C.A., Hasegawa K., Petrosino J.F., Piedra P.A., Sullivan A.F., Espinola J.A., Stewart C.J., Camargo C.A. (2020). Increased Moraxella and Streptococcus species abundance after severe bronchiolitis is associated with recurrent wheezing. J. Allergy Clin. Immunol..

[47] Segal L.N., Clemente J.C., Tsay J.C., Koralov S.B., Keller B.C., Wu B.G., Li Y., Shen N., Ghedin E., Morris A. (2016). Enrichment of the lung microbiome with oral taxa is associated with lung inflammation of a Th17 phenotype. Nat. Microbiol..

[48] Lehtinen M.J., Hibberd A.A., Mannikko S., Yeung N., Kauko T., Forssten S., Lehtoranta L., Lahtinen S.J., Stahl B., Lyra A. (2018). Nasal microbiota clusters associate with inflammatory response, viral load, and symptom severity in experimental rhinovirus challenge. Sci. Rep..

[49] De Maio F., Posteraro B., Ponziani F.R., Cattani P., Gasbarrini A., Sanguinetti M. (2020). Nasopharyngeal Microbiota Profiling of SARS-CoV-2 Infected Patients. Biol. Proced. Online.

[50] Harkema J.R., Carey S.A., Wagner J.G. (2006). The nose revisited: A brief review of the comparative structure, function, and toxicologic pathology of the nasal epithelium. Toxicol. Pathol..

[51] Önerci T.M. (2013). Nasal Physiology and Pathophysiology of Nasal Disorders.

[52] Negus V. (1958). Comparative Anatomy and Physiology of Nose and Paranasal Sinuses.

[53] Debertin A.S., Tschernig T., Tonjes H., Kleemann W.J., Troger H.D., Pabst R. (2003). Nasal-associated lymphoid tissue (NALT): Frequency and localization in young children. Clin. Exp. Immunol..

[54] Salzano F.A., Marino L., Salzano G., Botta R.M., Cascone G., D’Agostino Fiorenza U., Selleri C., Casolaro V. (2018). Microbiota Composition and the Integration of Exogenous and Endogenous Signals in Reactive Nasal Inflammation. J. Immunol. Res..

[55] Lee J.T., Frank D.N., Ramakrishnan V. (2016). Microbiome of the paranasal sinuses: Update and literature review. Am. J. Rhinol. Allergy.

[56] Cleland E.J., Drilling A., Bassiouni A., James C., Vreugde S., Wormald P.J. (2014). Probiotic manipulation of the chronic rhinosinusitis microbiome. Int. Forum Allergy Rhinol..

[57] Abreu N.A., Nagalingam N.A., Song Y., Roediger F.C., Pletcher S.D., Goldberg A.N., Lynch S.V. (2012). Sinus microbiome diversity depletion and Corynebacterium tuberculostearicum enrichment mediates rhinosinusitis. Sci. Transl. Med..

[58] Costantini C., Bellet M.M., Renga G., Stincardini C., Borghi M., Pariano M., Cellini B., Keller N., Romani L., Zelante T. (2020). Tryptophan Co-Metabolism at the Host-Pathogen Interface. Front. Immunol..

[59] Borghi M., Puccetti M., Pariano M., Renga G., Stincardini C., Ricci M., Giovagnoli S., Costantini C., Romani L. (2020). Tryptophan as a Central Hub for Host/Microbial Symbiosis. Int. J. Tryptophan Res..

[60] Cellini B., Zelante T., Dindo M., Bellet M.M., Renga G., Romani L., Costantini C. (2020). Pyridoxal 5′-Phosphate-Dependent Enzymes at the Crossroads of Host-Microbe Tryptophan Metabolism. Int. J. Mol. Sci..

[61] Platten M., Nollen E.A.A., Rohrig U.F., Fallarino F., Opitz C.A. (2019). Tryptophan metabolism as a common therapeutic target in cancer, neurodegeneration and beyond. Nat. Rev. Drug Discov..

[62] Munn D.H., Mellor A.L. (2013). Indoleamine 2,3 dioxygenase and metabolic control of immune responses. Trends Immunol..

[63] Grohmann U., Fallarino F., Puccetti P. (2003). Tolerance, DCs and tryptophan: Much ado about IDO. Trends Immunol..

[64] Shen B., Yi X., Sun Y., Bi X., Du J., Zhang C., Quan S., Zhang F., Sun R., Qian L. (2020). Proteomic and Metabolomic Characterization of COVID-19 Patient Sera. Cell.

[65] Thomas T., Stefanoni D., Reisz J.A., Nemkov T., Bertolone L., Francis R.O., Hudson K.E., Zimring J.C., Hansen K.C., Hod E.A. (2020). COVID-19 infection alters kynurenine and fatty acid metabolism, correlating with IL-6 levels and renal status. JCI Insight.

[66] Stockinger B., Di Meglio P., Gialitakis M., Duarte J.H. (2014). The aryl hydrocarbon receptor: Multitasking in the immune system. Annu. Rev. Immunol..

[67] Belladonna M.L., Orabona C. (2020). Potential Benefits of Tryptophan Metabolism to the Efficacy of Tocilizumab in COVID-19. Front. Pharmacol..

[68] Turski W.A., Wnorowski A., Turski G.N., Turski C.A., Turski L. (2020). AhR and IDO1 in pathogenesis of Covid-19 and the “Systemic AhR Activation Syndrome:” Translational review and therapeutic perspectives. Restor. Neurol. Neurosci..

[69] Giovannoni F., Li Z., Garcia C.C., Quintana F.J. (2020). A potential role for AHR in SARS-CoV-2 pathology. Preprint.

[70] Zelante T., Iannitti R.G., Cunha C., De Luca A., Giovannini G., Pieraccini G., Zecchi R., D’Angelo C., Massi-Benedetti C., Fallarino F. (2013). Tryptophan catabolites from microbiota engage aryl hydrocarbon receptor and balance mucosal reactivity via interleukin-22. Immunity.

[71] Scott S.A., Fu J., Chang P.V. (2020). Microbial tryptophan metabolites regulate gut barrier function via the aryl hydrocarbon receptor. Proc. Natl. Acad. Sci. USA.

[72] Powell D.N., Swimm A., Sonowal R., Bretin A., Gewirtz A.T., Jones R.M., Kalman D. (2020). Indoles from the commensal microbiota act via the AHR and IL-10 to tune the cellular composition of the colonic epithelium during aging. Proc. Natl. Acad. Sci. USA.

[73] Ji J., Qu H. (2019). Cross-regulatory Circuit Between AHR and Microbiota. Curr. Drug Metab..

[74] Agus A., Planchais J., Sokol H. (2018). Gut Microbiota Regulation of Tryptophan Metabolism in Health and Disease. Cell Host Microbe.

